# Hagfish slime exudate stabilization and its effect on slime formation and functionality

**DOI:** 10.1242/bio.025528

**Published:** 2017-06-15

**Authors:** L. J. Böni, R. Zurflüh, M. Widmer, P. Fischer, E. J. Windhab, P. A. Rühs, S. Kuster

**Affiliations:** 1 Department of Health Science and Technology, ETH Zürich, 8092 Zürich, Switzerland; 2 Department of Materials, ETH Zürich, 8093 Zürich, Switzerland

**Keywords:** Hagfish slime, Stabilization, Exudate storage, Vesicle rupture, Protein glue

## Abstract

Hagfish produce vast amounts of slime when under attack. The slime is the most dilute hydrogel known to date, and is a highly interesting material for biomaterial research. It forms from a glandular secrete, called exudate, which deploys upon contact with seawater. To study slime formation *ex vivo* and to characterize its material properties, stabilization of the sensitive slime exudate is crucial. In this study, we compared the two main stabilization methods, dispersion in high osmolarity citrate/PIPES (CP) buffer and immersion in oil, and tested the influence of time, temperature and pH on the stability of the exudate and functionality of the slime. Using water retention measurements to assess slime functionality, we found that CP buffer and oil preserved the exudate within the first 5 hours without loss of functionality. For longer storage times, slime functionality decreased for both stabilization methods, for which the breakdown mechanisms differed. Stabilization in oil likely favored temperature-sensitive osmotic-driven swelling and rupture of the mucin vesicles, causing the exudate to gel and clump. Extended storage in CP buffer resulted in an inhibited unraveling of skeins. We suggest that a water soluble protein glue, which mediates skein unraveling in functional skeins, denatures and gradually becomes insoluble during storage in CP buffer. The breakdown was accentuated when the pH of the CP buffer was raised from pH 6.7 to pH 8.5, probably caused by increased denaturation of the protein glue or by inferior vesicle stabilization. However, when fresh exudate was mixed into seawater or phosphate buffer at pH 6-9, slime functionality was not affected, showing pH insensitivity of the slime formation around a neutral pH. These insights on hagfish exudate stabilization mechanisms will support hagfish slime research at a fundamental level, and contribute to resolve the complex mechanisms of skein unraveling and slime formation.

## Abstract (precis)

**Summary:** Loss of functionality of hagfish slime exudate during storage is driven by mucin vesicle rupture when stabilized in oil and by gradually inhibited skein unraveling when stabilized in buffer.

## INTRODUCTION

**Fig. 1.**
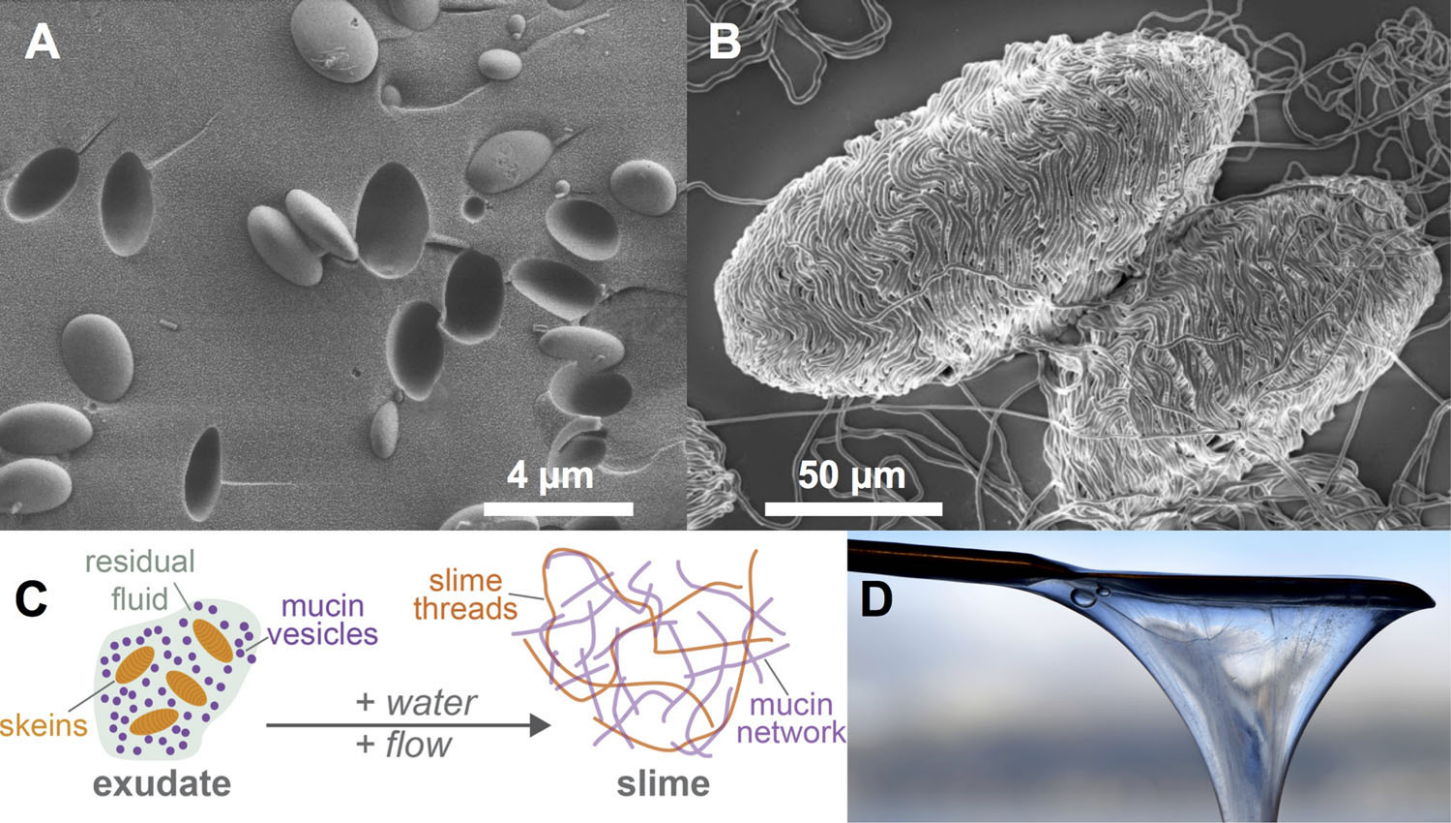
**Hagfish exudate and slime formation.** (A) Cryo-SEM image of hagfish mucin vesicles stabilized in heptane. (B) SEM image of hagfish thread skeins. (C) Schematic illustration of the slime formation mechanism. (D) Hagfish slime on a spatula.

Hagfish defend themselves against predators with vast amounts of slime (Zintzen et al., 2011). The slime forms when a glandular secrete, called exudate, is released from ventrolateral pores into the surrounding seawater. The exudate contains two major functional components, mucin vesicles (Fig. 1A) and thread skeins (Fig. 1B), as well as residual fluid, which is co-secreted with the skeins and vesicles. The contact with water combined with convective mixing (Winegard and Fudge, 2010) triggers the slime formation (Fig. 1C), causing the skeins to unravel and release their long, keratin-like threads. Simultaneously, the mucin vesicles swell and burst and release mucin-like glycoproteins, which interact with the threads and together form an ephemeral underwater network that physically entraps large amounts of water compared to other mucus hydrogels (Fig. 1D). The secreted slime is a unique biomaterial as it is the most dilute and fastest-forming hydrogel known to date (Fudge et al., 2005). The protein threads have similar properties to spider's silk (Fudge et al., 2003, 2005) and its viscosifying properties in extensional flow were suggested to be beneficial when attacked by suction feeders (Böni et al., 2016b). The slime threads are of high interest as they are flexible and tough, making them a promising source for novel fibers in clothing and biomedical applications. The hagfish mucins constitute a functional and relatively easily accessible source of mucins, which could be of particular interest for research on marine mucus or serve as a model for secreted mucin-like glycoproteins.

To obtain exudate from hagfish, the surrounding area of the slime pores is mildly electro-stimulated (Movie 1). The sensitive exudate is immediately stabilized either in a high osmolarity buffer or by immersion in oil. The latter method was first used by Ewoldt et al. (2011). The authors stabilized the exudate under mineral oil to study whole slime mechanics using rheology. Following a similar approach, medium-chain triglyceride (MCT) oil was found to have superior stabilization and handling properties to mineral oil (Böcker et al., 2016), mainly because handling with a micropipette is easier as the sample remains fluidized.

The stabilizing effect of a high osmolarity buffer was first described by Downing et al. (1981b), who found that exudate is stabilized in 1 M (NH_4_)_2_SO_4_. The stabilization criteria were further investigated by Salo et al. (1983), who reported that hagfish exudate is also stabilized in 0.5-1 M sodium citrate, as well as in 3 M sodium tartrate and sodium acetate, and were later systematically tested by Luchtel et al. (1991), who found that high osmolarity solutions of sulfate and phosphate were also able to stabilize the exudate. Piperazine-N,N′-bis(2-ethanesulfonic acid (PIPES, 0.1 M), PMSF (a serine protease inhibitor), as well as small amounts of EDTA, glycerol and MgCl_2_ were added to the stabilization buffer by Spitzer et al. (1988). A simpler version of this buffer (0.9 M citrate/0.1 M PIPES, pH 6.7), to which 0.02% NaN_3_, as well as a protease inhibitor cocktail, can be added to ensure bacterial and enzymatic stability (Böni et al., 2016b) became the widely used standard, largely favored by the facilitated separation of skeins and vesicles in citrate (Salo et al., 1983). The stabilization mechanism in buffer remains elusive, but the presence of high molarities of di- and trivalent anions was found to be important for stabilization of mucin vesicles. In addition, the stability criteria for skeins seem intimately linked to those of vesicles. The mucin vesicles are known to be stable for days in buffers with the appropriate ion composition and an osmolarity of ≥900 mOsmol/l (Luchtel et al., 1991), but thread skeins quickly lose their ability to unravel in CP buffer (Bernards et al., 2014). The effect of ionic strength and ionic composition needed for stabilization of hagfish exudate was studied in depth to stabilize mucin vesicles (Herr et al., 2010; Luchtel et al., 1991), but the influence of time, temperature and pH during storage of both main stabilization techniques was not investigated, although crucial for hagfish slime research, because stabilization of hagfish slime is directly linked to the complex slime formation mechanism.

In this study, we applied water retention measurement as an easy, robust and quantitative method to assess hagfish slime functionality, which is intimately linked to skein unraveling. This is of particular advantage when other techniques to gauge the material properties of fluids, gels and soft solids, such as rheology, are not applicable due to difficult sample handling, low torques or inhomogeneous structures. With this method, we investigate the impact of storage time, temperature and pH on exudate functionality, and discuss putative mechanisms which reduce or even lead to a loss of the slime-forming functionality, further on referred to as degradation. We found that exudate faces different pathways of degradation when stored in oil or in buffer. Furthermore, we provide evidence that the skeins of the Atlantic hagfish *(Myxine glutinosa)* contain a water-soluble glue (Bernards et al., 2014), which likely denatures during storage. We propose that the denatured glue is the main cause of ceased unraveling of hagfish skeins when stored in buffer over longer times. This work will support future research on hagfish slime by facilitating the choice of stabilization method, and by describing the degradation processes during storage.

## RESULTS AND DISCUSSION

### Water retention to assess hagfish slime functionality

**Fig. 2.**
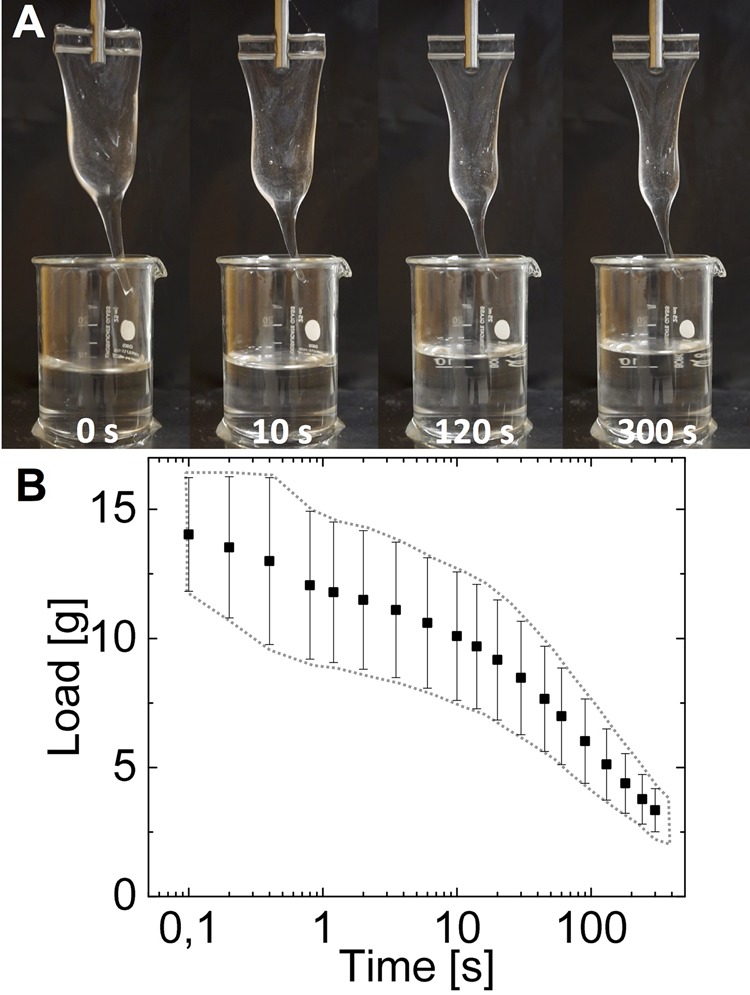
**Water retention of fresh hagfish slime in seawater at its natural concentration.** (A) Hagfish slime draining over 5 min. (B) Water retention of natural hagfish slime formed from fresh exudate.

Fresh unstabilized hagfish exudate at its natural concentration (∼0.02 wt%) (Böni et al., 2016a; Fudge et al., 2005) was able to gel the whole volume of 20 ml seawater (Fig. 2A), of which subsequently ∼14 ml (initial load, IL) could be lifted (Fig. 2B). After draining for ∼1 min under the influence of gravity, the slime lost around half its initial load. Once the water has drained, the slime can hardly rehydrate and the structure irreversibly collapses (Fig. S2) because the protein threads cluster and the slime loses its sieve-like structure (Fudge et al., 2005). The IL is a good measure of the effectiveness of water absorption and allows the convenient comparison of effects such as a varying exudate concentration or structure collapse (Fig. S2). Other authors similarly used a ‘removable mass’ to assess slime functionality and investigate the influence of dithiothreitol (DTT) (Koch et al., 1991) and stirring on slime formation (Lim et al., 2006), or measured the water egress from the slime to investigate the physical water entrapment and sieve-like properties (Fudge et al., 2005). The soft and wet character of biomaterials such as hagfish slime, in particular, often imposes substantial challenges to characterization methods applied in materials science (Ewoldt et al., 2014). Assessment of material properties of hagfish slime was so far mainly done by rheological measurements (Böcker et al., 2016; Böni et al., 2016a,b; Ewoldt et al., 2011), and by measuring the water egress (Böni et al., 2016a; Fudge et al., 2005). Although rheology allows the study of mechanics and flow behavior, the technique is limited by the softness of the slime and by potential inhomogeneities. Water retention measurements, in contrast, provide simple, but quantitative and robust information about the effectiveness of water uptake and the water-holding capacity, which are both critical criteria for hydrogel functionality. Water retention measurements therefore permit assessment of the functionality of hagfish slime, and the study of effects such as storage, which are otherwise hard to assess.

### Influence of stabilization method and time on slime functionality

**Fig. 3.**
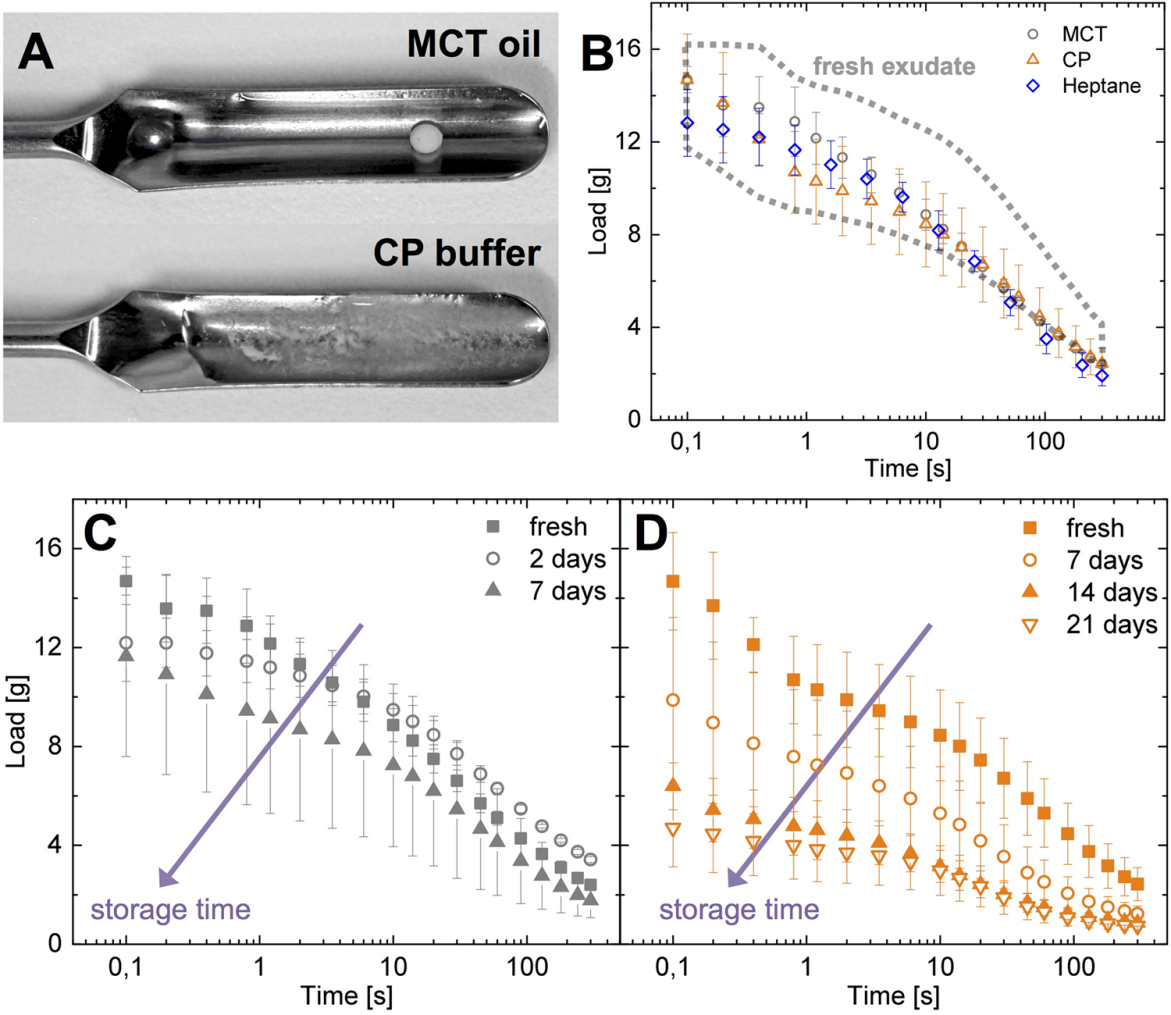
**Different hagfish exudate stabilization methods and their influence on slime functionality over time.** (A) Hagfish exudate stabilized in MCT oil and in CP buffer. (B) Comparison of water retention properties of slime formed from exudate stabilized in CP buffer, MCT oil and heptane after 5 h of storage to the water retention of fresh, unstabilized exudate (dashed region). (C,D) Influence of storage time on water retention of exudate stabilized in (C) MCT oil and (D) CP buffer.

Currently there are two main approaches to stabilize hagfish exudate: immersion in MCT oil and dispersion in a high osmolarity citrate/PIPES (CP) buffer (Fig. 3A). To evaluate if one method is superior to the other, water retention measurements were performed 5 hours after sampling (Fig. 3B). Both stabilization methods resulted in an almost indistinguishable slime functionality, as all possessed water retention properties similar to natural unstabilized hagfish slime. As a third stabilization method, heptane was tested, which also resulted in water retention properties similar to the other two methods. Heptane is intriguing due to its volatility, allowing removal of the stabilant at room temperature without leaving residues, which is not possible with MCT oil and CP buffer. Although handling was more difficult and stability lasted only ∼2 days, heptane is a promising method for short-term stabilization. In contrast to short-term stability, however, extended storage resulted in a loss of functionality and showed marked differences between the MCT oil and the CP buffer (Fig. 3C,D). Whereas MCT samples showed a rather sudden onset of functionality loss, the CP samples underwent a more gradual breakdown. After 5-10 days the MCT samples started to clump and did not deploy anymore when in water. The time for the onset was strongly dependent on each individual sample as often for biological samples, but also on the mechanical and temperature history of the sample. In contrast, exudate in CP buffer showed a gradual loss of functionality, which was found to be accompanied by a reduced skein unraveling. However, after a storage of 2 weeks the water retention properties markedly decreased, which could be linked to a reduced skein unraveling. From these observations it can be inferred that the principal mechanism of degradation is different for the stabilization methods. This hypothesis was further supported by the finding that the storage lifetime of a MCT sample could be extended when it was transferred to CP buffer before clumps occurred, giving it a second life. Stabilization and storage of slime exudate is crucial in hagfish slime research. We therefore studied the two putative degradation mechanisms by varying temperature and pH to determine the influence of storage on the exudate samples.

### Temperature induced mucin vesicle swelling and rupture

**Fig. 4.**
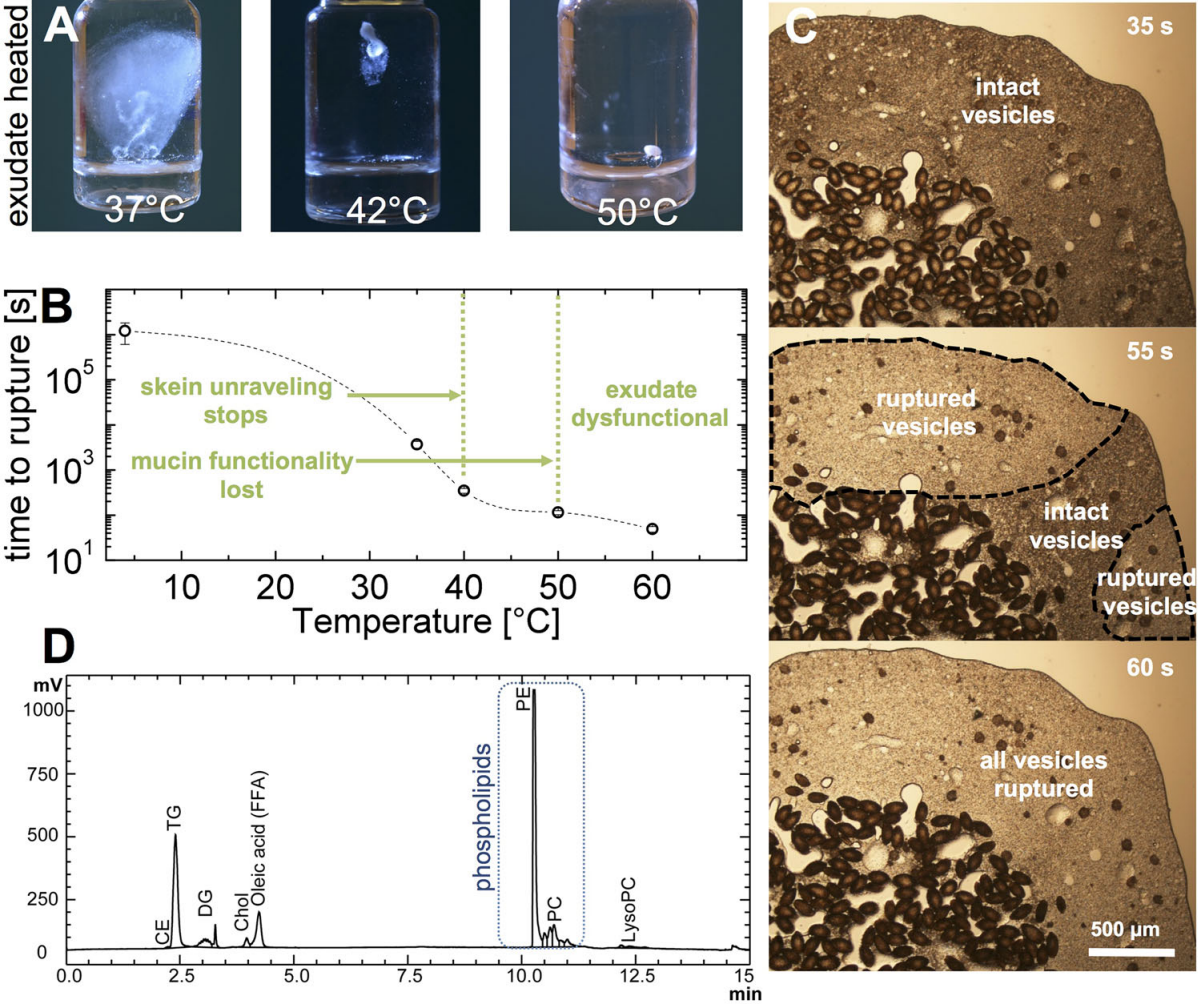
**Influence of temperature on hagfish exudate and slime functionality.** (A) Effect of heating MCT-stabilized exudate for 5 min on slime formation. (B) Hagfish mucin vesicle swelling and rupture time as a function of temperature. For vesicle rupture, MCT oil-stabilized exudate was used. The data point at 4°C refers to a sample stored in the fridge and indicates the time until clumping occurred in the fridge. The green text and the green dashed lines denote observations for exudate that was heated in CP buffer. The dashed line serves as a guide for the eye. (C) Microscopy image sequence of vesicle bursting at 60°C. The dashed areas outline the regions where mucin vesicles burst. (D) HPLC lipid analysis of hagfish mucin vesicles. CE, cholesteryl ester; TG, triglycerides; DG, diglycerides; Chol, Cholesterol; FFA, free fatty acid; PE, phosphatidylethanolamine; PC, phosphatidylcholine; LysoPC, lysophosphatidylcholine.

Exudate (20 μl covered with 200 μl MCT oil) was heated in an Eppendorf tube in a water bath to 37, 42 and 50°C for 5 min to test the influence of temperature on hagfish exudate stability and on slime formation (Fig. 4A). Whereas heating to 37°C had a minor impact, heating to 42 and 50°C resulted in almost completely inhibited slime formation. Heating MCT exudate under a microscope revealed that the sample gelled as soon as the heat wave reached the vesicles, suggesting that the vesicles swelled and ruptured at elevated temperatures (Fig. 4B,C; Movie 2). The ruptured vesicles released their mucin, forming a firm gel with the little available liquid originating from the residual fluid. The gel fails to swell rapidly when in water and also prevents a subsequent unraveling of the gel-embedded skeins. It is likely that the surrounding MCT oil favors swelling and rupture of the vesicles at elevated temperatures given their putative phospholipid bilayer membrane (Luchtel et al., 1991). To investigate the existence of a phospholipid bilayer, lipid analysis was performed (Fig. 4D). Substantial amounts of phosphatidylethanolamine (PE) and phosphatidylcholine (PC), and minor amounts of cholesterol (Chol) and lysophosphatidylcholine (LysoPC), were found, adding further evidence for the presence of a phospholipid membrane (Luchtel et al., 1991). A surrounding non-polar phase such as MCT oil likely favors an exchange of membrane phospholipids with the oil phase, leading to a break in vesicle membranes. This effect is more favored at higher temperatures (Hardy et al., 2013; Seantier et al., 2005), as membranes become softer when temperatures come close to the melting temperature of the lipids (Garcia-Manyes et al., 2005). The lipid analysis also showed the presence of triglycerides (TG), diglycerides (DG) and free fatty acids (FFA). Salo et al. (1983) found that hagfish mucin vesicles contain ∼4.8% lipids. As speculated by the authors, it is likely that non-membrane lipids are needed for the formation of a functional mucin gel. A positive effect of lipids on mucins is known as they can associate to mucins by hydrophobic interactions via the hydrophobic domains of mucin glycoproteins (Gong et al., 1990; Lai et al., 2009). Thereby, lipids were found to increase the viscosity and viscoelasticity of mucin (Lai et al., 2009; Zhang et al., 2015) and support their capability to form gels (Rogunova et al., 1997). The appearance of the heated and clumped sample was similar to old (>2 weeks) MCT samples, which were also gelled. However, the clump formation during storage in MCT oil is more likely to be a slow osmotic-driven swelling and rupture of mucin vesicles, given that the residual fluid of the exudate does not have obvious stabilizing effects on vesicles (Herr et al., 2010). It should be noted that clumping can also be induced when the samples are mechanically stressed, e.g. with a micropipette.

Vesicles stabilized in CP buffer do not rupture when mechanically stressed with a micropipette, when stored in the fridge for over 6 months or when heated to 50°C. The high ionic strength of the buffer likely keeps the glycoproteins in a stiff, condensed state, which makes merging of adjacent vesicles energetically unlikely. The polyanionic nature of mucins makes their conformation highly sensitive to ionic strength (Verdugo, 2012), which strongly reduces swelling of anionic gels at elevated salt levels (Khare and Peppas, 1995; Vasheghani-Farahani et al., 1990). Also, the high ionic strength of the CP buffer could stabilize the membrane due to a binding of the buffer ions on the phospholipids (Garcia-Manyes et al., 2005). Although the vesicular structure did not visually break at 50°C, the mucin probably denatured and aggregated inside the vesicle and lost the ability to swell, as the ability to form a viscous solution when mucin vesicles were mixed with water was lost. When the sample was heated to 42°C the vesicles were still able to form a viscous solution. Furthermore, heating exudate in CP to 42°C completely inhibited skein unraveling, which was not observed at 37°C. These results are in line with the findings of Bernards et al. (2014), who still observed unraveling at 35°C for *Eptatretus stoutii* skeins. Our observations show also that the unraveling of *M. glutinosa* skeins is highly temperature sensitive. We suggest that heat sensitivity of the skeins either originates from the presence of a protein glue that denatures at elevated temperatures and thus cannot mediate unraveling anymore, or that heat causes adjacent threads to stick together, thereby inhibiting unraveling.

### Influence of pH on storage and on slime formation

**Fig. 5.**
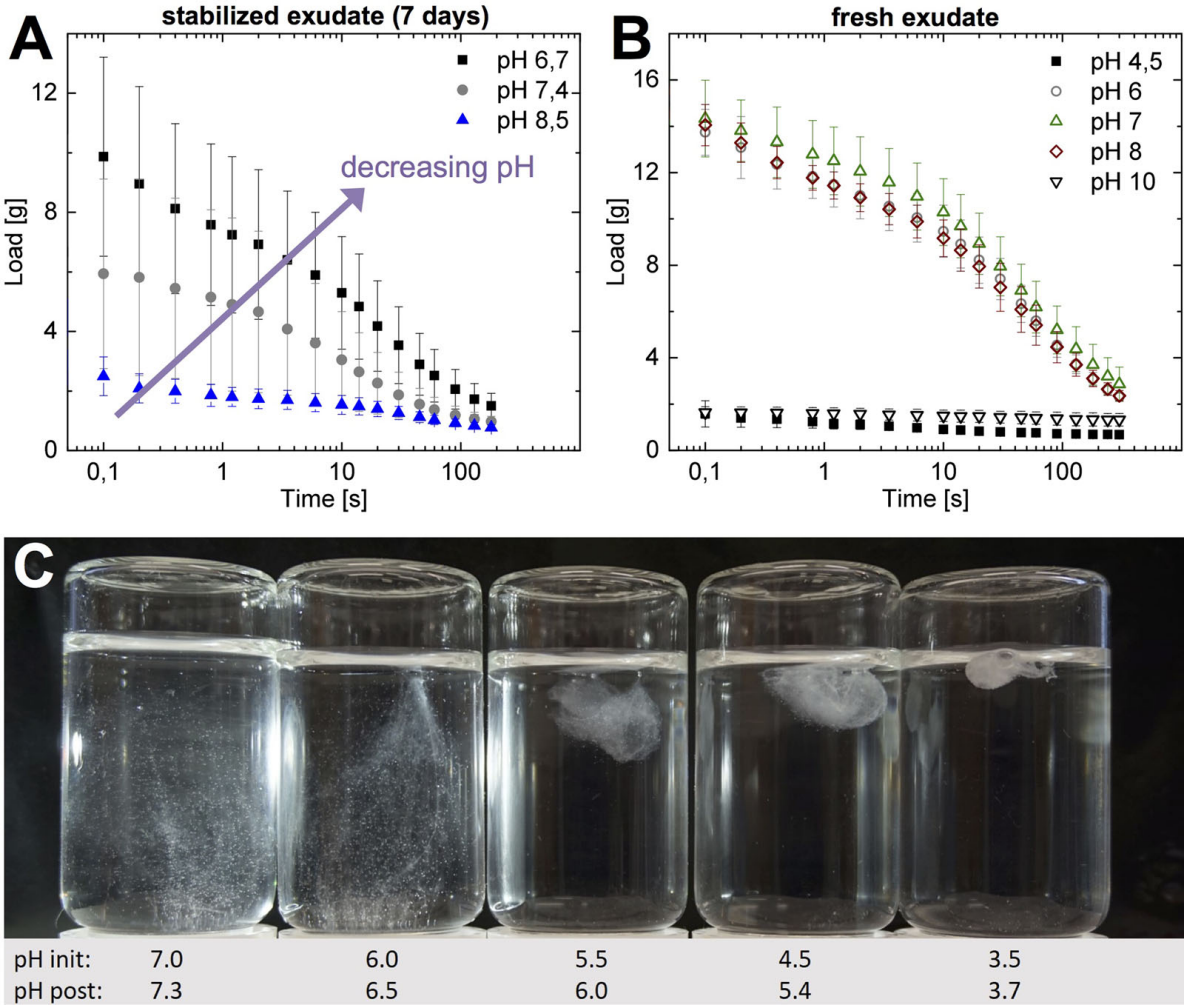
**Influence of pH on exudate storage and slime formation.** (A) Influence of pH of CP buffer on slime functionality (4 µl hagfish exudate from MCT oil was mixed into 200 µl CP buffer of different pH values and measured after 7 days of storage). (B) Water retention measurements of hagfish exudate from MCT oil in seawater at different pH values. (C) Hagfish exudate from MCT oil mixed into seawater at different pH values (pH init). The pH of the seawater slightly rose after the addition of hagfish exudate (pH post).

The pH of the CP buffer does not match the physiological pH values of hagfish blood (∼pH 8) (Wells et al., 1986), the residual fluid of the exudate (pH 7.3) (Herr et al., 2010), nor the pH of seawater (∼pH 8). We therefore investigated if an increased pH of the CP buffer has beneficial effects on storage (Fig. 5A). From the tested pH values (pH 8.5, 7.4 and 6.7), pH 6.7 (being the commonly used pH for CP buffer) showed the highest initial load, the highest degree of skein unraveling and thus the best slime functionality after 7 days of storage. In contrast, it was found that when fresh MCT oil-stabilized exudate was mixed into seawater or phosphate buffer between pH 6 and pH 9 (Fig. 5B,C; Fig. S3A), the slime showed similar water retentions, implying that the reduced functionality at higher pH originates from the storage. At pH extremes (pH 4.5 and pH 10) no functional slime formed. At low pH, the skeins did not unravel and aggregated with the mucins (Fig. S3B). At high pH, the mucins formed a tacky solution and many skeins did not unravel resulting in a weak network, which was unable to entrap water when lifted out of the water. These results show that skein unraveling is pH-dependent and that an increased pH of the CP buffer decreases the slime functionality by limiting the amount of unraveled skeins. We have two hypotheses as to why a lower pH stabilizes the exudate better. First, pH 6.7 and possibly even lower pH values close to pH 6 could be beneficial for mucin gel condensation inside the vesicle. Mucin granules are considered to have a low intraluminal pH (∼pH 6) (Ambort et al., 2012; Kesimer et al., 2010; Perez-Vilar et al., 2005). A low storage buffer pH, potentially close to the isoelectric point (pI) of mucin, is likely beneficial in keeping the mucin condensed. Close to the pI the number of counterions is minimal and therefore the osmotic pressure in and around the mucins decreases, resulting in a limited swelling capacity (Flory, 1953; Khare and Peppas, 1995)*.* A similar observation was made for mucin granules from human cervical cells, which showed a significantly decreased swelling velocity at pH 6.5 compared to pH 7.4 (Espinosa et al., 2002). Increasing the pH gradient from the CP buffer to the vesicle intragranular pH could thus result in compromised integrity of the mucin meshwork inside the granule (Perez-Vilar, 2007; Perez-Vilar et al., 2005) and cause partial swelling. These would inhibit vesicle swelling at the time of slime deployment and thus also limit skein unraveling, as both processes are suggested to be intimately linked (Luchtel et al., 1991; Winegard and Fudge, 2010). The second hypothesis is that a higher pH denatures the seawater soluble glue of the skeins faster. As most of the skeins are stripped from their plasma membrane when secreted through the slime pore (Downing et al., 1981a) they lack protection when exposed to the stabilization buffer. It is likely that the protein glue slowly denatures and becomes insoluble at elevated pH during storage.

### Extended storage in buffer denatures seawater soluble glue of thread skeins

**Fig. 6.**
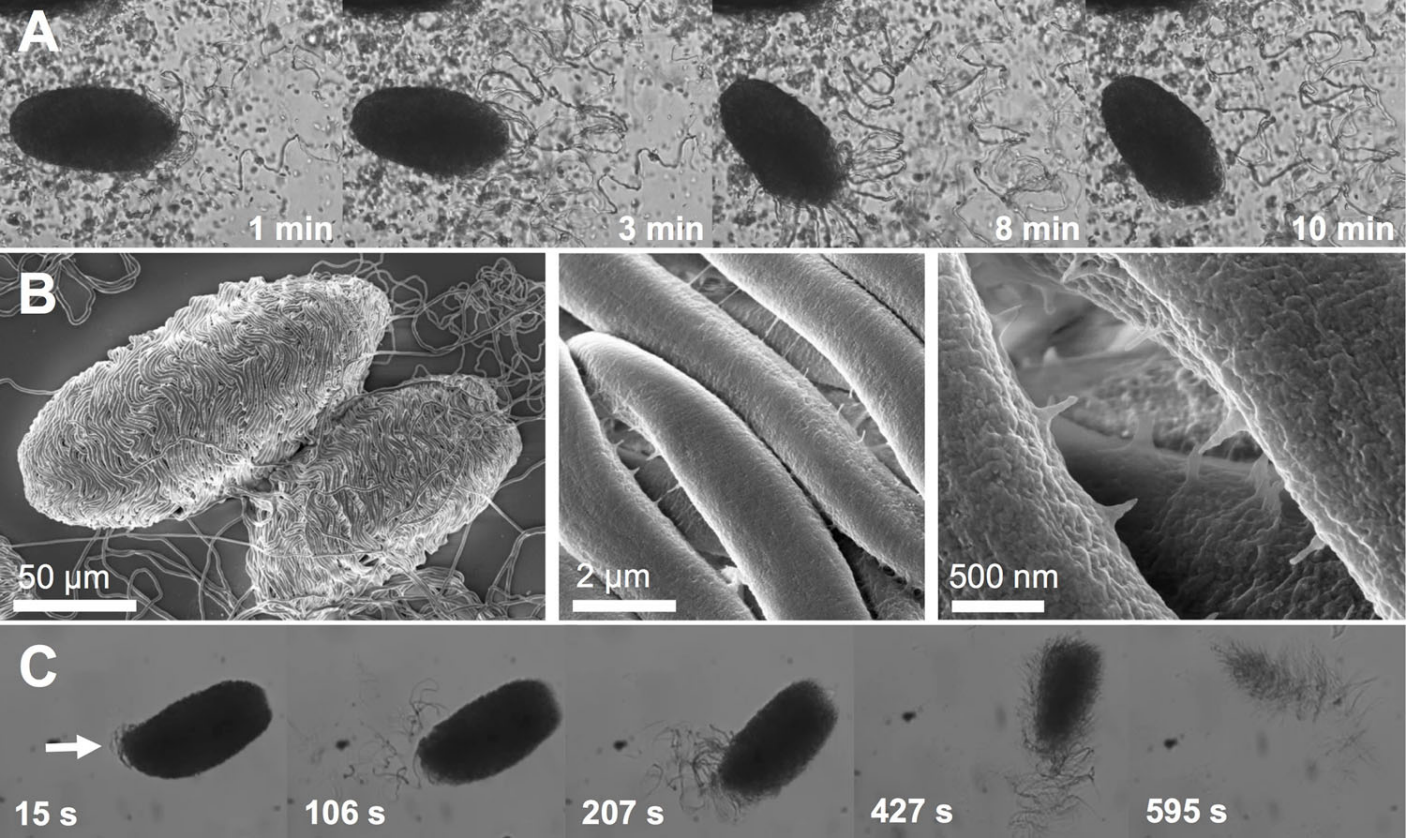
**Spontaneous unraveling of *M. glutinosa* skeins is mediated by a water-soluble protein, which becomes insoluble during storage in buffer.** (A) Spontaneous unraveling of a functional *M. glutinosa* skein surrounded by mucin vesicles in seawater in the absence of flow (MCT oil-stabilized sample). (B) SEM of *M. glutinosa* skeins that were unraveled with MilliQ water without stirring on a SEM slide (left). (Note that this image is reproduced from Fig. 1B.) The unfinished unraveling shows small bridging filaments between the thread parts (middle, closer view on right). (C) Old skein unravels in the presence of trypsin. The unraveling starts at the apical end of the skein (arrow).

MCT oil-stabilized hagfish skeins spontaneously unraveled in seawater (Fig. 6A; Movie 3). The absence of flow and the high local viscosity due to the concentrated mucin around the skeins likely slowed the unraveling down. The spontaneous unraveling suggests the existence of a seawater-soluble glue in *M. glutinosa* skeins, which dissolves and mediates unraveling by releasing the stored spring energy of the stored spring energy of the thread, as similarly observed for *E. stoutii* skeins (Bernards et al., 2014). Further evidence for the existence of a seawater soluble glue is provided in the SEM images in Fig. 6B. The images show a skein in the process of unraveling. In the inter-thread spacings, filamentous structures could be seen, which were also observed for *E. stoutii* skeins (Bernards et al., 2014). At high magnification the filamentous structures look like they are part of the rough surface of the skein, which is about to dissolve. Old skeins that were exposed to CP buffer for longer times fail to unravel in water, even in the presence of strong flow. However, when those old skeins were subjected to a trypsin solution they unraveled naturally similar to freshly harvested skeins, i.e. starting at the apical end of the skein (Fig. 6C; Movie 3) (Bernards et al., 2014). Similar to Bernards et al. (Bernards et al., 2014), we also observed that trypsin removed the glue faster than digesting the thread. We suggest that the protein glue denatures during storage and therefore becomes insoluble, being the main cause for a ceased unraveling after prolonged storage in CP buffer. Trypsin, cleaving on the C-terminal of lysine and arginine amino acid residues (Rodriguez et al., 2008) seems to be able to digest the denatured protein glue, thus re-initiating unraveling in water. It is possible that the glue is denatured by oxidation, by high ionic strength, or by other unfavorable conditions. The fact that elevated temperatures and non-physiological pH inhibit skein unraveling support the existence of a protein glue, as the protein is likely to denature. An alternative hypothesis is that adjacent loops of the thread stick to each other during storage, thereby inhibiting unraveling. The trypsin-driven unraveling would also support this mechanism as trypsin could digest the parts of the thread that are stuck together and liberate stored strain energy. Here, we note that our findings stand in slight contrast to the current theory of Winegard and Fudge (2010), who state that the unraveling of *M. glutinosa* skeins requires mucin strands and flow. Our movies revealed that seemingly neither flow nor mucin strands (the old skeins were washed with MilliQ and DTT prior to trypsin unraveling) are required for unraveling. We do not doubt the necessity of flow and mucin strands for the formation of a functional slime network, but we propose that flow and mucin strands are rather needed to propagate the spontaneous unraveling by mucin strands attaching to the unraveling threads and not for initialization of unraveling. However, further investigations are required to decouple the influence of single parameters such as flow, mucin and glue dissolution on skein unravelling, and on the complex mechanism of slime formation.

### Summary

In this study, the two main stabilization methods for hagfish exudate, immersion in MCT oil and dispersion in CP buffer, were compared. An overview of the major advantages and disadvantages of both stabilization methods are shown in Table 1.

**Table 1.**
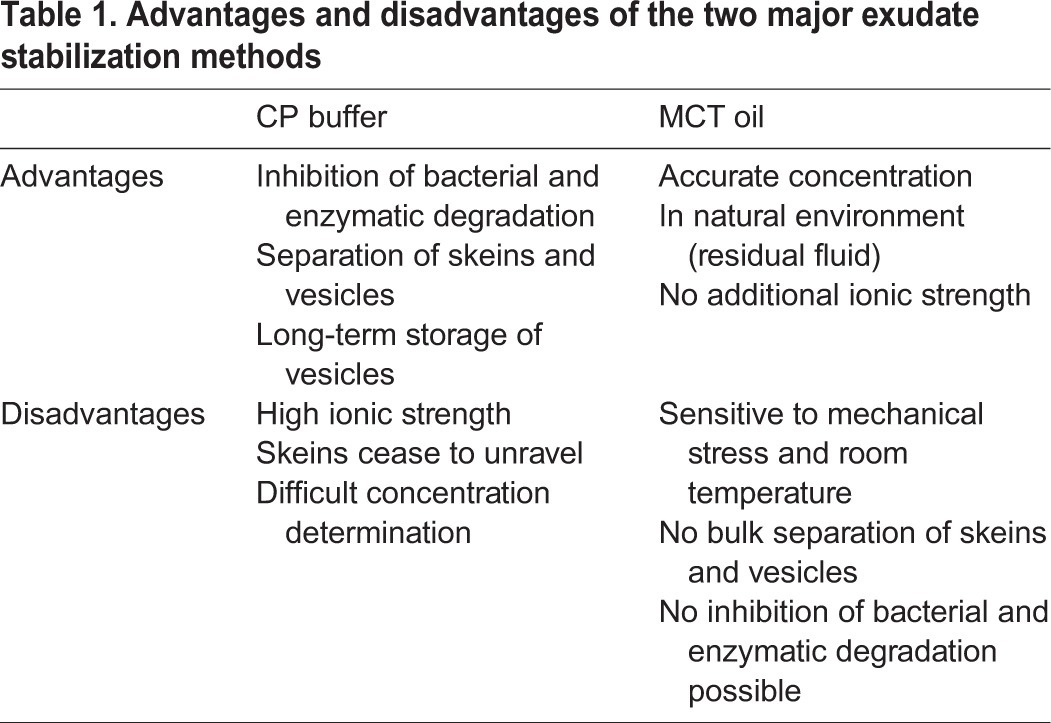
**Advantages and disadvantages of the two major exudate stabilization methods**

Using water retention measurements to assess the functionality of hagfish slime, it could be shown that for short storage times (<5 hours) both stabilization methods produced slime networks equal to fresh unstabilized hagfish exudate. As a novel stabilization method heptane was tested, which also preserved the functionality but was more difficult in handling than MCT oil as it had a drying effect on the exudate. Nevertheless, heptane could be used for applications where the stabilant is to be removed as it quickly evaporates. Therefore, depending on the purpose of exudate stabilization, a different stabilization technique has to be chosen.

Furthermore, we studied the boundary conditions necessary for a successful stabilization of exudate for laboratory experiments. Longer storage times caused the samples to degrade, and the MCT oil and CP buffer methods showed different breakdown mechanisms. MCT samples formed clumps after ∼5-7 days, probably due to osmotic driven swelling and rupture of the mucin vesicles. The mucin vesicles and the thread skeins were found to be highly sensitive to elevated temperatures, which possibly accelerates vesicle rupture. Rupture is likely favored by the non-stabilizing surrounding residual fluid and by phospholipids in the membrane, as they interact with the surrounding oil phase, causing leakage of the mucin and a subsequent gelation of the system. The gelled system does not form a slime anymore when in contact with seawater as the thread skeins are trapped in a dense mucin gel matrix. CP buffer-stabilized samples, on the other hand, showed a gradual loss of functionality over time, which could be linked to reduced skein unraveling. At long buffer exposure times, fewer skeins unraveled and therefore less water was retained. We propose that a seawater soluble glue, which holds the threads together and mediates unravelling, denatures during storage in the buffer and thus likely becomes insoluble. Evidence for the presence of such a glue as similarly observed for *E. stoutii* skeins (Bernards et al., 2014) was provided by trypsin-induced unraveling of old skeins as well as by electron microscopy images. The suggested increasing insolubility likely causes a gradual loss of skein unraveling and thus decreases slime functionality. When the pH of the CP buffer was raised from pH 6.7 to pH 8.5 the functionality further decreased. It could be shown that the negative influence of the higher pH was only observed after storage, but not when fresh exudate was mixed into seawater of higher pH. The reasons why a higher storage pH reduces slime functionality remain elusive, but it is likely that a higher pH denatures the water-soluble glue faster or has inferior stabilizing properties on the mucin vesicles.

Our findings underline the importance of rigorous cooling of MCT exudate samples and show that a stabilization buffer pH of 6.7 and possibly lower is beneficial to preserve the functionality of CP exudate samples. The observed degradation processes provide valuable guidelines for the choice of appropriate stabilization for hagfish exudate and will foster the uncovering of the complex mechanisms of slime formation.

## MATERIALS AND METHODS

### Exudate sampling and stabilization

Atlantic hagfish (*M. glutinosa*) were kindly provided by the Atlanterhavsparken in Ålesund, Norway. Sampling was performed according to the approved ethical application by the Forsøksdyrutvalget (FOTS ID 6912) based on a similar protocol to that described by Herr et al. (2010). In brief, hagfish were anaesthetized in fresh seawater using a 1:9 mixture of clove bud oil (Sigma-Aldrich) to ethanol at a concentration of 1 ml/l. Once sedated, the hagfish were transferred to a dissection tray and blotted dry. Slime exudate was obtained by mild electric stimulation (80 Hz, 18 V, HPG1, Velleman Instruments, Gavere, Belgium) on the ventral side (Movie 1). The released exudate was then collected and stabilized in MCT oil (Delios V, BASF, Ludwigshafen, Germany), heptane (VWR, Oslo, Norway), or in a high osmolarity CP buffer consisting of 0.9 M sodium citrate and 0.1 M PIPES at pH 6.7, 0.02% NaN_3_ (VWR, Oslo, Norway), and protease inhibitor (Sigmafast, Sigma-Aldrich). All samples were immediately stored at 4°C. The fish were transferred to a recovery bath after sampling. Import of the samples was approved by the Swiss Federal Food Safety and Veterinary Office (FSVO) and export was approved by the Norwegian Seafood Council.

### Water retention

The cohesiveness of hagfish slime mediated by its long fibers allows the lifting of the slime mass. The slime network formation efficiency can therefore be directly evaluated by lifting up the entire slime, as the network formation is based on the ability of the threads and mucins to form a cohesive slime. Slime for water retention measurements was prepared by placing MCT- or CP-stabilized exudate on the bottom of a 20 ml glass flask with a micropipette. Sterile filtered (0.2 μm PA-20/25 filter, Machery-Nagel, Düren, Germany) seawater from Norway was poured in and mixing was performed according to Ewoldt et al. (2011), by gently sloshing the water over the sample heads eight times. Water retention measurements were performed similar to described by Böni et al. (2016b). An in-house built mixing device (Fig. S1) was attached onto a laboratory scale and a video camera (Alpha 5100, Sony, Tokyo, Japan) was placed in front of the scale to optically monitor the weight change over time. The mixed slime was transferred from the glass flask to a small beaker and placed on the scale, and then the mixing device lowered into the slime and rotated 10 times to wrap up the slime mass. The wrapped slime was then lifted, arrested in the upper position, and the water egress was recorded gravimetrically. The exudate concentration of the measurements was determined according to the assumption of Ewoldt et al. (2011) (density of the exudate is close to 1 g/ml, as ∼66% of the exudate mass is water). MCT oil samples could be accurately pipetted with a micropipette as the exudate sank to the bottom of the Eppendorf tube (Movie 1). To ensure an accurate concentration of exudate in CP buffer, exudate from MCT oil was pipetted into a defined volume of CP buffer for stability and pH tests. For pH experiments, the pH of seawater was adjusted with HCl and NaOH, respectively. All experiments were performed in triplicate.

### Microscopy

Light microscopy images were captured on a Nikon Diaphot (Nikon, Tokyo, Japan) and analyzed with the NIS elements D3.0 software. Rupture of mucin vesicles at different temperatures was investigated by mounting a heating plate onto the microscope. The heating plate was preheated to the corresponding temperature for 5 min. A microscopy slide with 1 μl exudate from MCT oil and covered with a coverslip was mounted on the heating plate. The time needed for the vesicle halo to burst was used as rupture time. All experiments were performed in triplicate. The value for rupture at 4°C was determined by measuring the time needed for MCT samples to clump in the fridge. Skein unraveling with trypsin (from porcine pancreas, Sigma-Aldrich; 30,000 units/ml in 5 mM potassium phosphate buffer, pH 6.5) was performed on old skeins (8 months) that were stabilized in CP buffer and did not unravel anymore. The skeins were washed with 100 mM DTT to remove mucins and then dialyzed against MilliQ water (24 h, 3×) and freeze dried. Non-freeze-dried skeins were also investigated and showed the same unraveling behavior.

### SEM

For scanning electron microscopy (SEM), fresh unstabilized exudate was placed on SEM plates and a few drops of MilliQ water were added onto the sample without stirring. The samples were air dried for 48 h and subsequently sputter coated (Bal-Tec SCD 050 sputter coater, Leica Microsystems, Wetzlar, Germany) with a 3 nm thick platinum layer. SEM was performed on a LEO 1530 (Carl-Zeiss SMT AG, Oberkochen, Germany). For cryo-SEM, heptane-stabilized exudate samples were snap frozen with a high pressure freezer (Bal-Tec HPM100, Leica Microsystems) and subsequently freeze fractured (Bal-Tec BAF060, Leica Microsystems). SEM was conducted on a LEO 1530.

### Lipid analysis

Vesicles for lipid analysis were obtained similar to the protocol of Salo et al. (1983). Exudate stabilized in CP buffer was filtered through a series (60 and 20 μm) of nylon mesh filters (Merck, Darmstadt, Germany) to separate the mucin vesicles from the skeins. The vesicle solution was washed three times with CP buffer and subsequently concentrated by repeated centrifugation (2960 ***g*** for 30 min). The concentrated vesicles were then decomposed according to the protocol of Bligh and Dyer (1959). A mixture of chloroform methanol, chloroform and nanopure water, at a ratio of 2:2:1, were added to the sample while homogenizing with a colloidal mill (Ultra Turrax, IKA, Staufen im Breisgau, Germany). The homogenized sample was centrifuged (2960 ***g*** for 10 min), the chloroform phase was collected, and the contained lipids were concentrated by bubbling N_2_ through the sample. Lipid classes were qualitatively analyzed by a Nexera/Prominence HPLC system (Shimadzu, Kyoto, Japan) coupled to a LTII evaporative light scattering detector (ELSD, Shimadzu) according to Olsson et al. (2012) with the following modifications. The sample was dissolved in a mixture of eluents (eluent A:eluent B=1:1 by vol.) to a 0.05-0.10 mg/ml concentration and separated on Reprosil-Pur 120 CN column (250×4.6 mm, 5 µm) equipped with a guard column (ReproSil-Pur 120 CN, 10×4.6 mm, 5 µm) (Dr Maisch GmbH, Ammerbuch, Germany) kept at a constant temperature (26.0°C). A binary gradient elution at a constant flow rate of 1.0 ml/min, and consisting of eluents A=*n*-hexane and B=toluene:methanol:acetic acid:triethylamine=60:40:0.2:0.1 (by wt) was used with the following timetable: at 0.0-3.0 min 95:5 (%A:%B), at 8.0 min 60:40, at 14.0 min 50:50, at 15.0 min 5:95. The ELSD evaporation temperature was 30°C and the gas pressure (N_2_) was kept at 3.20 bar. The injection volume was 10 µl. All solvents were of LC grade (Merck and VWR). The lipid classes were identified by comparison with retention times of commercial analytical standards (Avanti, NuCheck and Sigma-Aldrich).

## References

[BIO025528C1] Ambort D., Johansson M. E. V., Gustafsson J. K., Nilsson H. E., Ermund A., Johansson B. R., Koeck P. J. B., Hebert H. and Hansson G. C. (2012). Calcium and pH-dependent packing and release of the gel-forming MUC2 mucin. *Proc. Natl. Acad. Sci. USA* 109, 5645-5650. 10.1073/pnas.1120269109 22451922 PMC3326483

[BIO025528C2] Bernards M. A. Jr, Oke I., Heyland A. and Fudge D. S. (2014). Spontaneous unraveling of hagfish slime thread skeins is mediated by a seawater-soluble protein adhesive. *J. Exp. Biol.* 217, 1263-1268. 10.1242/jeb.096909 24744422

[BIO025528C3] Bligh E. G. and Dyer W. J. (1959). A rapid method of total lipid extraction and purification. *Can. J. Biochem. Physiol.* 37, 911-917. 10.1139/o59-099 13671378

[BIO025528C4] Böcker L., Rühs P. A., Böni L., Fischer P. and Kuster S. (2016). Fiber-enforced hydrogels: hagfish slime stabilized with biopolymers including κ-carrageenan. *ACS Biomater. Sci. Eng.* 2, 90-95. 10.1021/acsbiomaterials.5b00404 33418646

[BIO025528C5] Böni L., Rühs P. A., Windhab E. J., Fischer P. and Kuster S. (2016a). Gelation of soy milk with hagfish exudate creates a flocculated and fibrous emulsion- and particle gel. *PLoS ONE* 11, e0147022 10.1371/journal.pone.0147022 26808048 PMC4726539

[BIO025528C6] Böni L., Fischer P., Böcker L., Kuster S. and Rühs P. A. (2016b). Hagfish slime and mucin flow properties and their implications for defense. *Sci. Rep.* 6, 30371 10.1038/srep30371 27460842 PMC4961968

[BIO025528C7] Downing S. W., Spitzer R. H., Salo W. L., Downing J. S., Saidel L. J. and Koch E. A. (1981a). Threads in the hagfish slime gland thread cells: organization, biochemical features, and length. *Science* 212, 326-328. 10.1126/science.212.4492.326 17792088

[BIO025528C8] Downing S. W., Salo W. L., Spitzer R. H. and Koch E. A. (1981b). The hagfish slime gland: a model system for studying the biology of mucus. *Science* 214, 1143-1145. 10.1126/science.7302586 7302586

[BIO025528C9] Espinosa M., Noé G., Troncoso C., Ho S. B. and Villalón M. (2002). Acidic pH and increasing [Ca(2+)] reduce the swelling of mucins in primary cultures of human cervical cells. *Hum. Reprod.* 17, 1964-1972. 10.1093/humrep/17.8.1964 12151422

[BIO025528C10] Ewoldt R. H., Winegard T. M. and Fudge D. S. (2011). Non-linear viscoelasticity of hagfish slime. *Int. J. Non Linear Mech.* 46, 627-636. 10.1016/j.ijnonlinmec.2010.10.003

[BIO025528C11] Ewoldt R. H., Johnston M. T. and Caretta L. M. (2015). Experimental challenges of shear rheology: how to avoid bad data. In Complex Fluids in Biological Systems: Experiment, Theory, and Computation (ed. S. E. Spagnolie), pp. 207-241. New York, NY: Springer 10.1007/978-1-4939-2065-5_6

[BIO025528C12] Flory P. J. (1953). *Principles of Polymer Chemistry*. Ithaca, NY: Cornell University Press.

[BIO025528C13] Fudge D. S., Gardner K. H., Forsyth V. T., Riekel C. and Gosline J. M. (2003). The mechanical properties of hydrated intermediate filaments: insights from hagfish slime threads. *Biophys. J.* 85, 2015-2027. 10.1016/S0006-3495(03)74629-3 12944314 PMC1303373

[BIO025528C14] Fudge D. S., Levy N., Chiu S. and Gosline J. M. (2005). Composition, morphology and mechanics of hagfish slime. *J. Exp. Biol.* 208, 4613-4625. 10.1242/jeb.01963 16326943

[BIO025528C15] Garcia-Manyes S., Oncins G. and Sanz F. (2005). Effect of temperature on the nanomechanics of lipid bilayers studied by force spectroscopy. *Biophys. J.* 89, 4261-4274. 10.1529/biophysj.105.065581 16150966 PMC1366991

[BIO025528C16] Gong D. H., Turner B., Bhaskar K. R. and Lamont J. T. (1990). Lipid binding to gastric mucin: protective effect against oxygen radicals. *Am. J. Physiol.* 259, G681-G686. 2221078 10.1152/ajpgi.1990.259.4.G681

[BIO025528C17] Hardy G. J., Nayak R. and Zauscher S. (2013). Model cell membranes: techniques to form complex biomimetic supported lipid bilayers via vesicle fusion. *Curr. Opin. Colloid Interface Sci.* 18, 448-458. 10.1016/j.cocis.2013.06.004 24031164 PMC3767439

[BIO025528C18] Herr J. E., Winegard T. M., O'Donnell M. J., Yancey P. H. and Fudge D. S. (2010). Stabilization and swelling of hagfish slime mucin vesicles. *J. Exp. Biol.* 213, 1092-1099. 10.1242/jeb.038992 20228345

[BIO025528C19] Kesimer M., Makhov A. M., Griffith J. D., Verdugo P. and Sheehan J. K. (2010). Unpacking a gel-forming mucin: a view of MUC5B organization after granular release. *Am. J. Physiol. Lung Cell. Mol. Physiol.* 298, L15-L22. 10.1152/ajplung.00194.2009 19783639 PMC2806194

[BIO025528C20] Khare A. R. and Peppas N. A. (1995). Swelling/deswelling of anionic copolymer gels. *Biomaterials* 16, 559-567. 10.1016/0142-9612(95)91130-Q 7492721

[BIO025528C21] Koch E. A., Spitzer R. H., Pithawalla R. B. and Downing S. W. (1991). Keratin-like components of gland thread cells modulate the properties of mucus from hagfish (Eptatretus stouti). *Cell Tissue Res.* 264, 79-86. 10.1007/BF00305724 1711418

[BIO025528C22] Lai S. K., Wang Y.-Y., Wirtz D. and Hanes J. (2009). Micro- and macrorheology of mucus. *Adv. Drug Deliv. Rev.* 61, 86-100. 10.1016/j.addr.2008.09.012 19166889 PMC2736374

[BIO025528C23] Lim J., Fudge D. S., Levy N. and Gosline J. M. (2006). Hagfish slime ecomechanics: testing the gill-clogging hypothesis. *J. Exp. Biol.* 209, 702-710. 10.1242/jeb.02067 16449564

[BIO025528C24] Luchtel D. L., Martin A. W. and Deyrup-Olsen I. (1991). Ultrastructure and permeability characteristics of the membranes of mucous granules of the hagfish. *Tissue Cell* 23, 939-948. 10.1016/0040-8166(91)90043-S 18621196

[BIO025528C25] Olsson P., Holmbäck J. and Herslöf B. (2012). Separation of lipid classes by HPLC on a cyanopropyl column. *Lipids* 47, 93-99. 10.1007/s11745-011-3627-0 22101922

[BIO025528C26] Perez-Vilar J. (2007). Mucin granule intraluminal organization. *Am. J. Respir. Cell Mol. Biol.* 36, 183-190. 10.1165/rcmb.2006-0291TR 16960124 PMC2176109

[BIO025528C27] Perez-Vilar J., Olsen J. C., Chua M. and Boucher R. C. (2005). pH-dependent intraluminal organization of mucin granules in live human mucous/goblet cells. *J. Biol. Chem.* 280, 16868-16881. 10.1074/jbc.M413289200 15718243

[BIO025528C28] Rodriguez J., Gupta N., Smith R. D. and Pevzner P. A. (2008). Does trypsin cut before proline? *J. Proteome Res.* 7, 300-305. 10.1021/pr0705035 18067249

[BIO025528C29] Rogunova M., Blackwell J., Jamieson A., Pasumarthy M. and Gerken T. (1997). Effect of lipids on the structure and rheology of gels formed by canine submaxillary mucin. *Biorheology* 34, 295-308. 10.1016/S0006-355X(98)00006-7 9578805

[BIO025528C30] Salo W. L., Downing S. W., Lidinsky W. A., Gallagher W. H., Spitzer R. H. and Koch E. A. (1983). Fractionation of hagfish slime gland secretions: partial characterization of the mucous vesicle fraction. *Prep. Biochem.* 13, 103-135. 10.1080/00327488308068743 6878177

[BIO025528C31] Seantier B., Breffa C., Félix O. and Decher G. (2005). Dissipation-enhanced quartz crystal microbalance studies on the experimental parameters controlling the formation of supported lipid bilayers. *J. Phys. Chem. B* 109, 21755-21765. 10.1021/jp053482f 16853826

[BIO025528C32] Spitzer R. H., Koch E. A. and Downing S. W. (1988). Maturation of hagfish gland thread cells: Composition and characterization of intermediate filament polypeptides. *Cell Motil. Cytoskeleton* 11, 31-45. 10.1002/cm.970110105 2463104

[BIO025528C33] Vasheghani-Farahani E., Vera J. H., Cooper D. G. and Weber M. E. (1990). Swelling of ionic gels in electrolyte solutions. *Ind. Eng. Chem. Res.* 29, 554-560. 10.1021/ie00100a010

[BIO025528C34] Verdugo P. (2012). Supramolecular dynamics of mucus. *Cold Spring Harb. Perspect. Med.* 2, a009597 10.1101/cshperspect.a009597 23125200 PMC3543103

[BIO025528C35] Wells R. M., Forster M. E., Davison W., Taylor H. H., Davie P. S. and Satchell G. H. (1986). Blood oxygen transport in the free-swimming hagfish, Eptatretus cirrhatus. *J. Exp. Biol.* 123, 43-53. 3746198 10.1242/jeb.123.1.43

[BIO025528C36] Winegard T. M. and Fudge D. S. (2010). Deployment of hagfish slime thread skeins requires the transmission of mixing forces via mucin strands. *J. Exp. Biol.* 213, 1235-1240. 10.1242/jeb.038075 20348334

[BIO025528C37] Zhang J., Lv Y., Wang B., Zhao S., Tan M., Lv G. and Ma X. (2015). Influence of microemulsion-mucin interaction on the fate of microemulsions diffusing through pig gastric mucin solutions. *Mol. Pharm.* 12, 695-705. 10.1021/mp500475y 25608210

[BIO025528C38] Zintzen V., Roberts C. D., Anderson M. J., Stewart A. L., Struthers C. D. and Harvey E. S. (2011). Hagfish predatory behaviour and slime defence mechanism. *Sci. Rep.* 1, 131 10.1038/srep00131 22355648 PMC3216612

